# Excessive Cytokine Response to Rapid Proliferation of Highly Pathogenic Avian Influenza Viruses Leads to Fatal Systemic Capillary Leakage in Chickens

**DOI:** 10.1371/journal.pone.0068375

**Published:** 2013-07-09

**Authors:** Saya Kuribayashi, Yoshihiro Sakoda, Takeshi Kawasaki, Tomohisa Tanaka, Naoki Yamamoto, Masatoshi Okamatsu, Norikazu Isoda, Yoshimi Tsuda, Yuji Sunden, Takashi Umemura, Noriko Nakajima, Hideki Hasegawa, Hiroshi Kida

**Affiliations:** 1 Laboratory of Microbiology, Department of Disease Control, Graduate School of Veterinary Medicine, Hokkaido University, Sapporo, Japan,; 2 Research Office Concerning the Health of Human and Birds, Abashiri, Japan,; 3 Laboratory of Comparative Pathology, Department of Veterinary Clinical Sciences, Graduate School of Veterinary Medicine, Hokkaido University, Sapporo, Japan,; 4 Department of Pathology, National Institute of Infectious Diseases, Tokyo, Japan,; 5 Research Center for Zoonosis Control, Hokkaido University, Sapporo, Japan; Virginia Polytechnic Institute and State University, United States of America

## Abstract

Highly pathogenic avian influenza viruses (HPAIVs) cause lethal infection in chickens. Severe cases of HPAIV infections have been also reported in mammals, including humans. In both mammals and birds, the relationship between host cytokine response to the infection with HPAIVs and lethal outcome has not been well understood. In the present study, the highly pathogenic avian influenza viruses A/turkey/Italy/4580/1999 (H7N1) (Ty/Italy) and A/chicken/Netherlands/2586/2003 (H7N7) (Ck/NL) and the low pathogenic avian influenza virus (LPAIV) A/chicken/Ibaraki/1/2005 (H5N2) (Ck/Ibaraki) were intranasally inoculated into chickens. Ty/Italy replicated more extensively than Ck/NL in systemic tissues of the chickens, especially in the brain, and induced excessive mRNA expression of inflammatory and antiviral cytokines (IFN-γ, IL-1β, IL-6, and IFN-α) in proportion to its proliferation. Using *in situ* hybridization, IL-6 mRNA was detected mainly in microglial nodules in the brain of the chickens infected with Ty/Italy. Capillary leakage assessed by Evans blue staining was observed in multiple organs, especially in the brains of the chickens infected with Ty/Italy, and was not observed in those infected with Ck/NL. In contrast, LPAIV caused only local infection in the chickens, with neither apparent cytokine expression nor capillary leakage in any tissue of the chickens. The present results indicate that an excessive cytokine response is induced by rapid and extensive proliferation of HPAIV and causes fatal multiple organ failure in chickens.

## Introduction

Influenza A virus infections are found in a variety of birds and mammals, including humans. These viruses are classified into subtypes on the basis of the antigenic specificity of the surface glycoproteins hemagglutinin (HA) and neuraminidase (NA). To date, viruses of 16 HA subtypes (H1-H16) and 9 NA subtypes (N1-N9) have been isolated from avian species [1–3]. Avian influenza viruses causing 75% or greater mortality in chickens within 10 days after intravenous inoculation are categorized as highly pathogenic avian influenza viruses (HPAIVs) [4]. The clinical course of HPAIV infection in chickens varies between virus strains. Chickens infected with HPAIVs generally show ruffled feathers, depression, and edema of the face, comb, and wattles; they develop subcutaneous hemorrhages in unfeathered skin and die within a few days [5]. In some acute cases of the disease, chickens infected with HPAIV suddenly die while sleeping, without showing apparent clinical signs or gross lesions [6,7].

Cleavage activation of the HA by host proteases is required for influenza A virus replication. Among HPAIVs, the HAs have multiple basic amino acid residues at their cleavage sites, which permit cleavage activation by ubiquitous proteases such as furin and PC6, leading to systemic infection in chickens [8,9]. On the other hand, the HAs of low pathogenic avian influenza viruses (LPAIVs) are cleaved only by trypsin-like proteases expressed in the respiratory and intestinal tracts, leading to local infection in chickens. Although cleavage activation of the HA is *sine qua non* for the pathogenicity of HPAIV, host factors involved in the pathogenesis of HPAIV infection have not been identified.

Cytokines are regulators of the host response to infection, inflammation, trauma and immunity. In the course of infection, the balance of inflammatory and anti-inflammatory cytokines is important for induction of a proper immune response, clearance of pathogen, and healing. In particular, inflammatory cytokines such as IL-1β, IL-6, and IFN-γ mediate rubor, fever, pain, vascular permeability, and cellular infiltration [10]. Excessive cytokine responses to various pathogens are harmful to the host and have also been found in cases of lethal infection with HPAIV in humans, mice, ferrets, and monkeys [11–13]. Extensive viral proliferation and high levels of cytokines and chemokines, including IP-10, MCP-1, IL-8, IL-6, TNF-α, and IL-10, were found in the sera and lungs of humans and experimental animals infected with H5N1 influenza virus [14–16]. Although this aberrant cytokine response is assumed to cause acute respiratory distress syndrome and death in mammals, H5 HPAIV infection was also lethal to mice lacking TNF and IL-1 receptors, and immunosuppressive treatment was not always an effective therapy for H5 HPAIV infection in mice [17,18]. High expression of cytokines such as IL-6, IL-12, and IL-18 was observed in the lungs and spleen of the chickens infected with H5 HPAIV, and a large amount of type I IFN was also detected in tissues and plasma of the chickens infected with H5 HPAIV [19–22]. However, in per-acute cases of infection with another H5 HPAIV, cytokine mRNA expression was not significantly increased in the lungs of chickens until death [22]. In both mammals and birds, the relationship between pathogenesis of influenza virus infections and host responses are not well understood.

In the present study, to assess the role of host cytokines in the pathogenesis of avian influenza, two H7 HPAIVs and an LPAIV were intranasally inoculated into chickens and viral proliferation, mRNA expression of cytokines, and capillary permeability were analyzed during the early stages of infection. Our findings demonstrate that systemic vascular disorder induced by excessive cytokine response to HPAIV proliferation is critical for the pathogenesis of highly pathogenic avian influenza in chickens. 

## Materials and Methods

### Viruses

The 2 HPAIVs A/turkey/Italy/4580/1999 (H7N1) (Ty/Italy) [23] and A/chicken/Netherlands/2586/2003 (H7N7) (Ck/NL) [24] were kindly provided by Dr. I. Capua of Instituto Zooprofilattico Sperimentale delle Venezie (Legnaro, Padova, Italy). The intravenous pathogenicity index (IVPI) and the 50% chicken lethal dose of Ty/Italy after intranasal inoculation were 3.00 and 10^2.0^ [50% egg infectious dose (EID_50_)], whereas those of Ck/NL were 2.68 and 10^5.8^ EID_50_, respectively. The LPAIV A/chicken/Ibaraki/1/2005 (H5N2) (Ck/Ibaraki) [25] was kindly provided by Dr. S. Yamaguchi, National Institute of Animal Health (Tsukuba, Ibaraki, Japan), and the IVPI was 0.00 [26]. Viruses were propagated in 10-day-old embryonated chicken eggs at 35°C for 40–48 h.

### Experimental infection of chickens with influenza viruses

In brief, 10^6.0^ EID_50_ of Ty/Italy or Ck/NL were intranasally inoculated into eight 4-week-old chickens (Boris brown, Hokuren, Hokkaido, Japan). The chickens were observed every day until 12 days post inoculation (dpi). Sera from the surviving chickens were examined using a hemagglutination-inhibition (HI) test [27] at 12 dpi.

To examine the proliferation of each virus and the host cytokine response during the early stages of infection, 10^6.0^ EID_50_ of Ty/Italy, Ck/NL, or Ck/Ibaraki were each intranasally inoculated into groups of twelve 4-week-old chickens (Boris brown and Juria, Hokkaido Chuo Shukeijo Corporation, Hokkaido, Japan). Three chickens per group were euthanized, and the brains, lungs, and spleens were collected at 24, 48, 72, and 96 hours post inoculation (hpi). At 48 and 96 hpi, the tissue specimens were soaked in 10% formalin for histopathological analysis. To determine virus infectivity titers, the tissues were homogenized using a Multi-Beads Shocker (Yasui Kikai, Osaka, Japan) and were suspended in minimum essential medium (Nissui, Tokyo, Japan) containing 100,000 U/ml penicillin (Meiji Seika, Tokyo, Japan), 10 mg/ml streptomycin (Meiji Seika), 0.3 mg/ml gentamicin (Schering-Plough, Osaka, Japan), 0.2% nystatin (Shigma-Aldrich, Missouri, U.S.A.), and 0.5% bovine serum albumin fraction V (Roche Diagnostics, Mannheim, Germany). These suspensions and peripheral blood were serially diluted with PBS, inoculated into 10-day-old embryonated chicken eggs, and incubated at 35°C for 48 h. Virus titers were determined according to the Reed and Munch method [28] and were expressed as EID_50_ per gram of tissue or milliliter of blood. For quantitative real-time PCR analysis of the mRNA expression of cytokines, a portion of each tissue was soaked in RNAlater (Ambion, Texas, U.S.A.) and stored at −20°C.

All animals were housed in self-contained units (Tokiwa Kagaku, Tokyo, Japan) at the BSL-3 facility of the Graduate School of Veterinary Medicine, Hokkaido University, Japan. The institutional animal care and use committee of the Graduate School of Veterinary Medicine approved these animal experiments (approval numbers: 1051, 1112), and all experiments were performed under the guidance of the Institute for Laboratory Animal Research (ILAR).

### RNA isolation and quantitative real-time PCR

Total RNA was extracted from each tissue using the RNeasy Mini Kit (QIAGEN, Maryland, U.S.A.) according to the manufacturer’s instructions. To remove genomic DNA, total RNA was treated with DNase I (QIAGEN). One microgram of total RNA per sample was reverse-transcribed with Oligo (dT)_15_ primers, RNase inhibitor (Invitrogen, California, U.S.A.), and M-MLV reverse transcriptase (Invitrogen). The reaction mixtures comprised 2 µl of cDNA, 10 µl of Light Cycler 480 SYBR Green I master mix (Roche Diagnostic) or KAPA SYBR Fast qPCR master mix (KAPA, Boston, U.S.A.), 2 µl of forward and reverse primer (10 µM), and 4 µl of pure water. Reactions were carried out on a Light Cycler 480 System II (Roche Diagnostic). The primers were as follows: β-actin (forward: 5′-CTG TTC GCC TTT CAG ACC TAC A-3′, reverse: 5′-CAT GGT GAT TTT CTC TAT CCA GTC C-3′) (Accession number: NM_205518), IFN-α [29], TNF-α [30], IFN-γ, IL-1β, and IL-6 [31]. The copy number of cytokine mRNA was normalized to that of β-actin, and the data was shown as mean fold change compared with that of uninfected control birds.

### Immunohistochemistry and *in situ* hybridization

Tissues were fixed with 10% formalin and embedded in paraffin and sectioned at 3–4 µm. For light microscopy, the sections were subjected to hematoxylin–eosin staining. To detect viral antigens, the sections were stained using the streptavidin–biotin–immunoperoxidase complex method with the Histofine SAB-PO (M) kit (Nichirei Biosciences, Tokyo, Japan) according to the manufacturer’s instructions. The sections were deparaffinized and digested with 0.1% trypsin at 37°C for 30 min, and endogenous peroxidase activity was quenched with 3% H_2_O_2_ in methanol. After blocking of nonspecific reactions with normal goat serum, the sections were incubated with mouse anti-NP monoclonal antibody (produced in our laboratory; 1: 1,000) at 4°C for 12 h. The chromomeric reaction was carried out by incubating the sections in 0.05 M Tris-HCl buffer containing 0.02% 3,3′-diaminobenzidinetetrahydrochloride (Dojindo Laboratories, Kumamoto, Japan), 0.005% H_2_O_2_, and 0.01 M imidazol (Sigma), and the sections were counterstained with Mayer’s hematoxylin.

To detect IL-6 mRNA, *in situ* hybridization was performed using a QuantiGene viewRNA Tissue Assay (Affymetrix, California, U.S.A.). The viewRNA probe set consisted of 18 probes designed to cover 1,142 base pairs of avian IL-6 mRNA sequence (Accession number: MN_204628). In brief, after deparaffinization, brain sections from infected and uninfected chickens were treated with a target retrieval solution (DAKO, California, U.S.A.) at 95°C for 40 min, and with 0.1 µg/ml of proteinase K (DAKO) at 37°C for 15 min, as described previously [32]. They were subsequently incubated with a viewRNA probe at 40°C for 2 h. After washing 3 times in wash buffer, hybridization with PreAmplifier Mix QT (Affymetrix), Amplifier Mix QT (Affymetrix), and Label Probe 1 conjugated with alkaline phosphatase (Affymetrix) was performed according to the manufacturer’s instructions. After incubation of FastRed substrate (Warp Red Chromogen Kit, Biocare Medical, California, U.S.A.), the slides were counterstained with Gill’s hematoxylin.

### Evaluation of the integrity of blood tissue barriers

Capillary permeability was evaluated by extravasation with Evans blue (EB; Wako), as described previously [33]. The infected chickens and normal chickens (n = 5) were intravenously injected with 25 mg/kg of 2% EB dye in sterile saline at 4 days after infection with Ty/Italy (n = 6), Ck/NL (n = 5), or Ck/Ibaraki (n = 5). After 3 h, the chickens were exsanguinated and brains, lungs, hearts, spleens, kidneys, and colons were collected. Portions of the tissues were soaked in 500 µl of formamide at 38°C for 24 h. The amount of EB in supernatants was measured against a standard of 90% formamide in saline at 630 nm using a Model 680 Microplate Reader (Bio-Rad Laboratories, California, U.S.A.), and EB levels (ng/g) were calculated for each tissue using a standard curve.

### Statistical analysis

Statistical analyses of results were made using unpaired, parametric or non-parametric Student’s *t*-test. Differences were considered statistically significant when P < 0.05. 

## Results

### Pathogenicity of H7 HPAIVs in chickens

**Figure 1:**
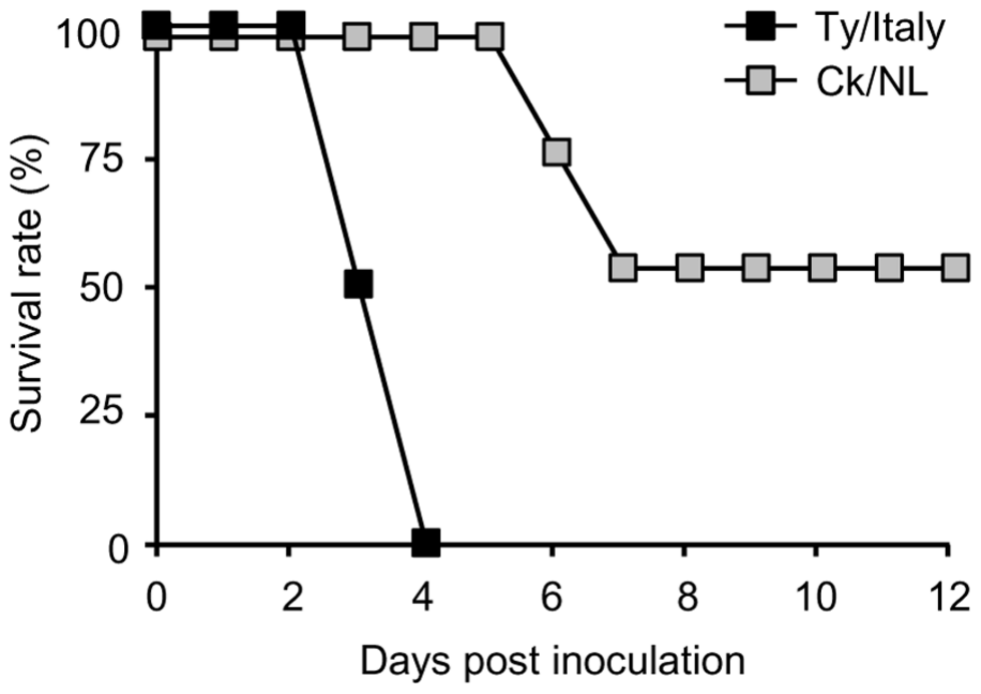
Survival rates of the chickens inoculated with Ty/Italy or Ck/NL. Chickens were intranasally inoculated with 10^6.0^ EID_50_ of Ty/Italy or Ck/NL and were observed for 12 days.

To compare the pathogenicity of Ty/Italy and CK/NL in chickens, 10^6.0^ EID_50_ of each virus was inoculated intranasally into chickens. All the chickens inoculated with Ty/Italy showed lethargy, inner hemorrhage of the unfeathered skin, edema of the face and legs, and red conjunctiva from 2–3 dpi. These symptoms worsened rapidly, and all chickens died by 4 dpi (Figure 1). On the other hand, in the chickens infected with Ck/NL, symptoms appeared at 3–4 dpi and half of the chickens died at 6–7 dpi; and the others survived for the 12 observation days. HI antibodies against homologous virus antigens were detected in the sera of the surviving chickens at titers of 512–1,024.

### Proliferation of HPAIVs and LPAIV in chickens

**Figure 2:**
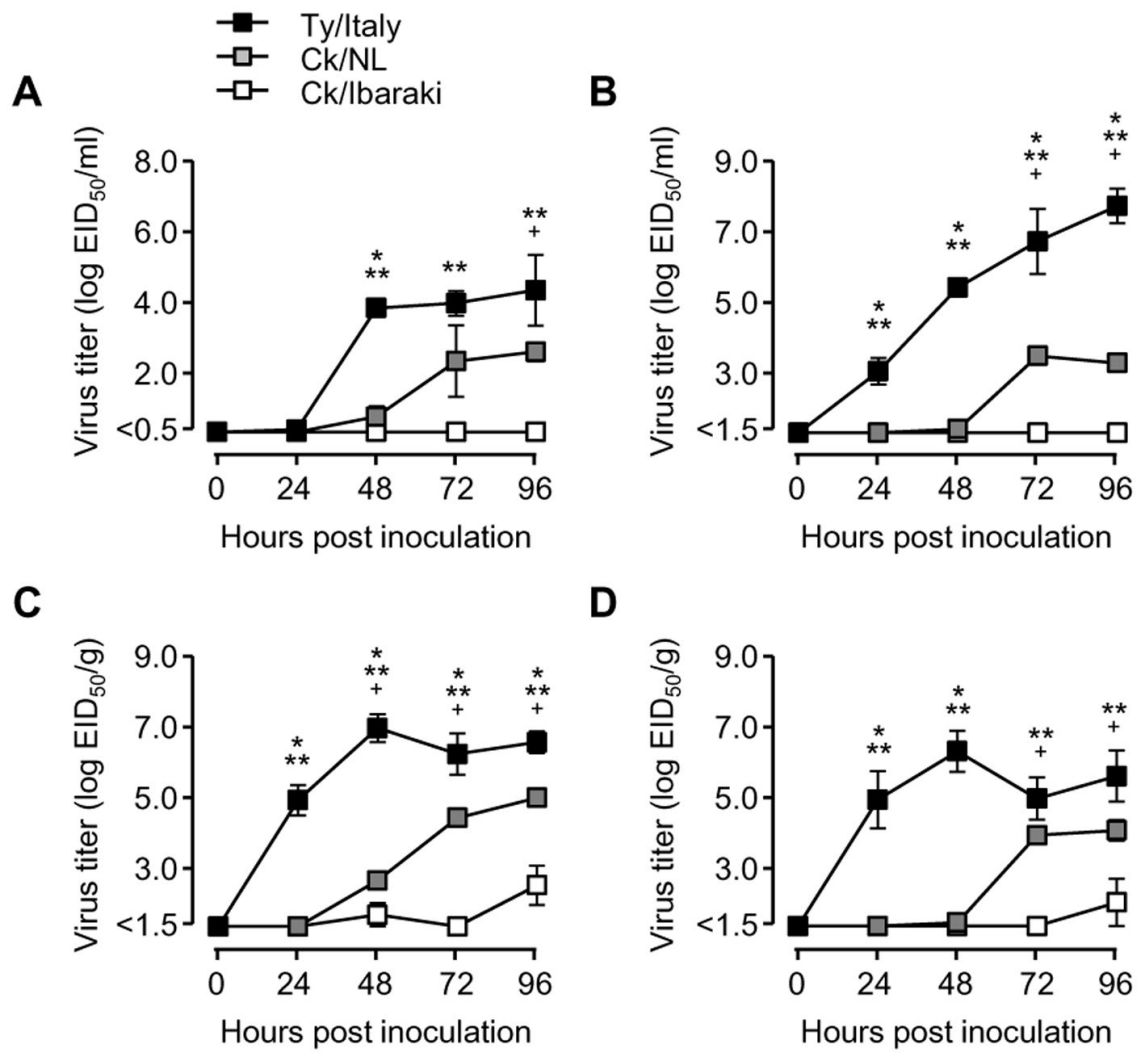
Comparison of HPAIVs and LPAIV proliferation. After 10^6.0^ EID_50_ of Ty/Italy, Ck/NL, or Ck/Ibaraki was intranasally inoculated into chickens, peripheral blood (**A**), brains (**B**), lungs (**C**), and spleens (**D**) were collected every 24 h, and infectivity titers were determined by inoculation of 10-day-old embryonated eggs. The mean values with corresponding standard errors from 3 chickens are shown. * *p*<0.05 between Ty/Italy and Ck/NL, ** *p*<0.05 between Ty/Italy and Ck/Ibaraki, + *p*<0.05 between Ck/NL and Ck/Ibaraki.

To compare the proliferation of Ty/Italy, Ck/NL, and Ck/Ibaraki in the chickens, infectivity titers in the peripheral blood, brains, lungs, and spleens of the chickens inoculated with each of the 3 viruses were determined at 24, 48, 72, and 96 hpi (Figure 2A–D). The Ty/Italy strain rapidly and extensively replicated in all tissues examined, and the highest infectivity titers were 10^7.0^ to 10^8.0^ EID_50_/g in the brain at 72–96 hpi. In contrast, Ck/NL replicated more slowly than Ty/Italy in all the chicken tissues, with infectivity titers 10^2^-10^4^ times lower than those of Ty/Italy. Infectious viruses in the tissues of the chickens inoculated with Ck/NL gradually decreased at 120 and 144 hpi (data not shown). Disease signs appeared in the chickens inoculated with Ty/Italy at 48 hpi, and 1 bird died at 3 dpi and another at 4 dpi. The chickens inoculated with Ck/NL showed mild disease signs after 72 hpi. On the other hand, no chicken inoculated with Ck/Ibaraki showed any disease signs, and only a small amount of viruses were occasionally recovered from their lungs (Figure 2C) and spleens (Figure 2D) at 48 and 96 hpi (≦10^1.8^ to 10^3.7^ EID_50_). At 120 and 144 hpi, viruses were not recovered from the chickens inoculated with Ck/Ibaraki (data not shown). These 3 avian influenza viruses showed different patterns of virulence, and severe disease signs were accompanied by rapid viral proliferation in the chickens.

### Expression of cytokine mRNA in the tissues of chickens inoculated with HPAIVs or LPAIV

**Figure 3:**
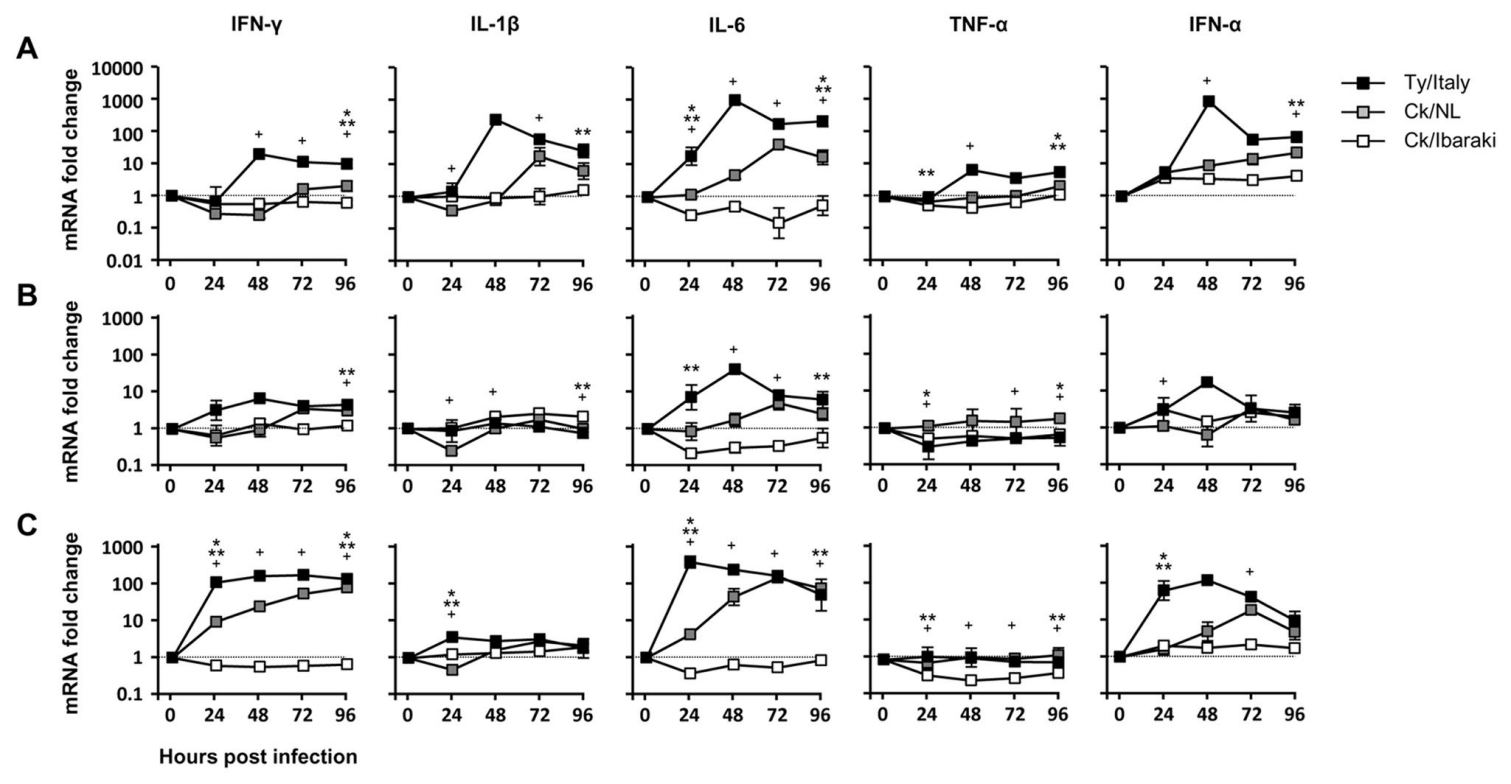
Comparison of cytokine mRNA expression. Tissues were collected from 3 chickens per group every 24 h after inoculation with 10^6.0^ EID_50_ of HPAIVs or LPAIV, and cytokine mRNA expression in the brain (**A**), lungs (**B**), and spleen (**C**) was analyzed using real-time PCR. Data are expressed as mean fold changes with standard errors relative to β-actin mRNA. Data of dead chickens was eliminated from the results. * *p*<0.05 between Ty/Italy and Ck/NL, ** *p*<0.05 between Ty/Italy and Ck/Ibaraki, + *p*<0.05 between Ck/NL and Ck/Ibaraki.

To examine cytokine responses to infection with each virus, the mRNA expression of the inflammatory cytokines IFN-γ, IL-1β, IL-6, and TNF-α and the antiviral cytokine IFN-α was determined in the brains, lungs, and spleens of the chickens inoculated with Ty/Italy, Ck/NL, or Ck/Ibaraki, respectively (Figure 3A–C). Strong or moderate expression of cytokines was observed in the tissues of the chickens infected with Ty/Italy or Ck/NL, respectively. In particular, cytokine expression was markedly elevated in brains (Figure 3A). The highest cytokine expression was found at 48 hpi in the brains of the chickens inoculated with Ty/Italy, with increase in IFN-γ, IL-1β, IL-6, TNF-α, and IFN-α levels by 21-, 286-, 1,179-, 7.0-, and 1,032-fold, respectively, compared to those of uninfected chickens. Cytokines were also strongly expressed in the brain at 96 hpi with Ty/Italy. In the brains of the chickens inoculated with Ck/NL, IL-1β, IL-6, and IFN-α levels were increased by 28-, 49-, and 25-fold, respectively, at 72–96 hpi. The levels of IFN-γ and TNF-α were not significantly changed after infection with Ck/NL. In the lungs (Figure 3B) and spleens (Figure 3C), the expression patterns of cytokines were similar to those in the brain. The largest increase in mRNA expression was observed in the lungs of the chickens inoculated with Ty/Italy or Ck/NL, with 6.6- or 3.5-fold, 38- or 5.6-fold, and 16- or 6.3-fold increase in IFN-γ, IL-6, and IFN-α, respectively. In the spleens of the chickens infected with Ty/Italy or Ck/NL, IFN-γ, IL-6, and IFN-α levels were increased by 132- or 58-fold, 276- or 103-fold, and 98- or 15-fold, respectively. The increases of IL-1β and TNF-α levels were not apparent in the lungs and spleens of the chickens infected with Ty/Italy nor Ck/NL. It is noteworthy that mRNA expression of IL-6 was most apparent among the 4 cytokines in each tissue of the chickens infected with HPAIVs. In contrast, the level of cytokine expression was not significantly high in each tissue of the chickens infected with Ck/Ibaraki (≦3.5-fold). Thus, excessive cytokine responses were observed in the chickens infected with HPAIVs.

### Distribution of virus antigen in the brain

**Figure 4:**
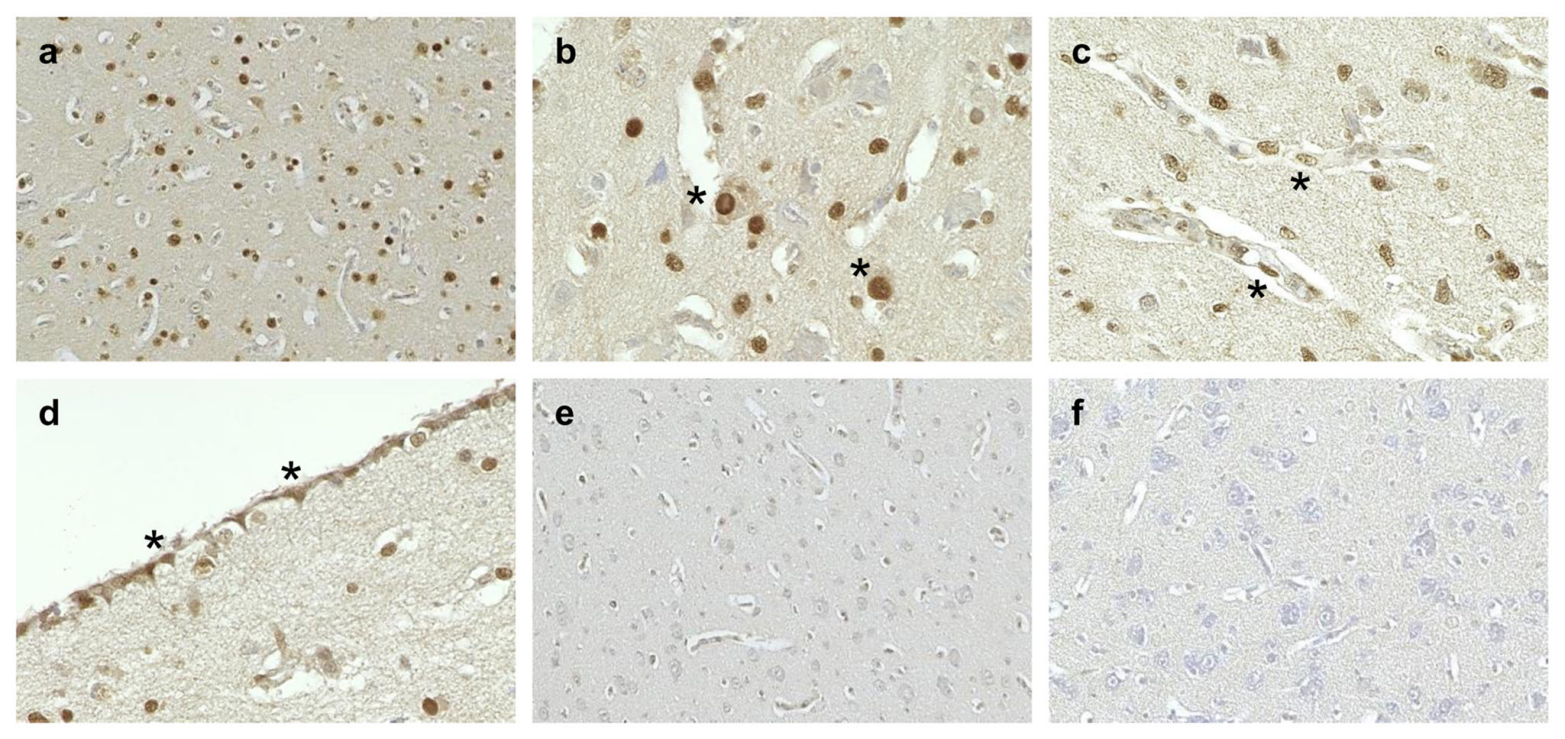
Immunohistochemical analysis of viral antigens in the brain. Viral antigen (brown signals) was detected in the Ty/Italy-infected chickens at 96 hpi (**a**). Antigen-positive cells included neuronal cells (asterisks in **b**), endothelial cells (asterisks in **c**), and ependymal cells (asterisks in **d**). Virus antigen-positive cells were not detected in the brains of the Ck/NL- (**e**) nor Ck/Ibaraki-infected chickens (**f**) at 96 hpi. Original magnification, ×100: a, e, f; ×400: b, c, d.

To investigate the distribution of virus antigen in the brains of the virus-infected chickens, immunohistochemistry was performed on brain sections at 48 and 96 hpi. The large amount of antigen was detected throughout the cerebrum of Ty/Italy-infected chickens at 96 hpi (Figure 4a). Antigen-positive cells included neurons, ependymal cells, astrocytes, oligodendrocytes, microglial, endothelial, and necrotic cells (Figure 4b–d). In Ty/Italy-infected chickens, antigen was detected on the brain section at 48 hpi. In contrast, viral antigen-positive cells were not detected in the brains of the chickens infected with Ck/NL nor Ck/Ibaraki at 48 and 96 hpi (Figure 4e and f).

### Distribution of IL-6 mRNA in the brain

**Figure 5:**
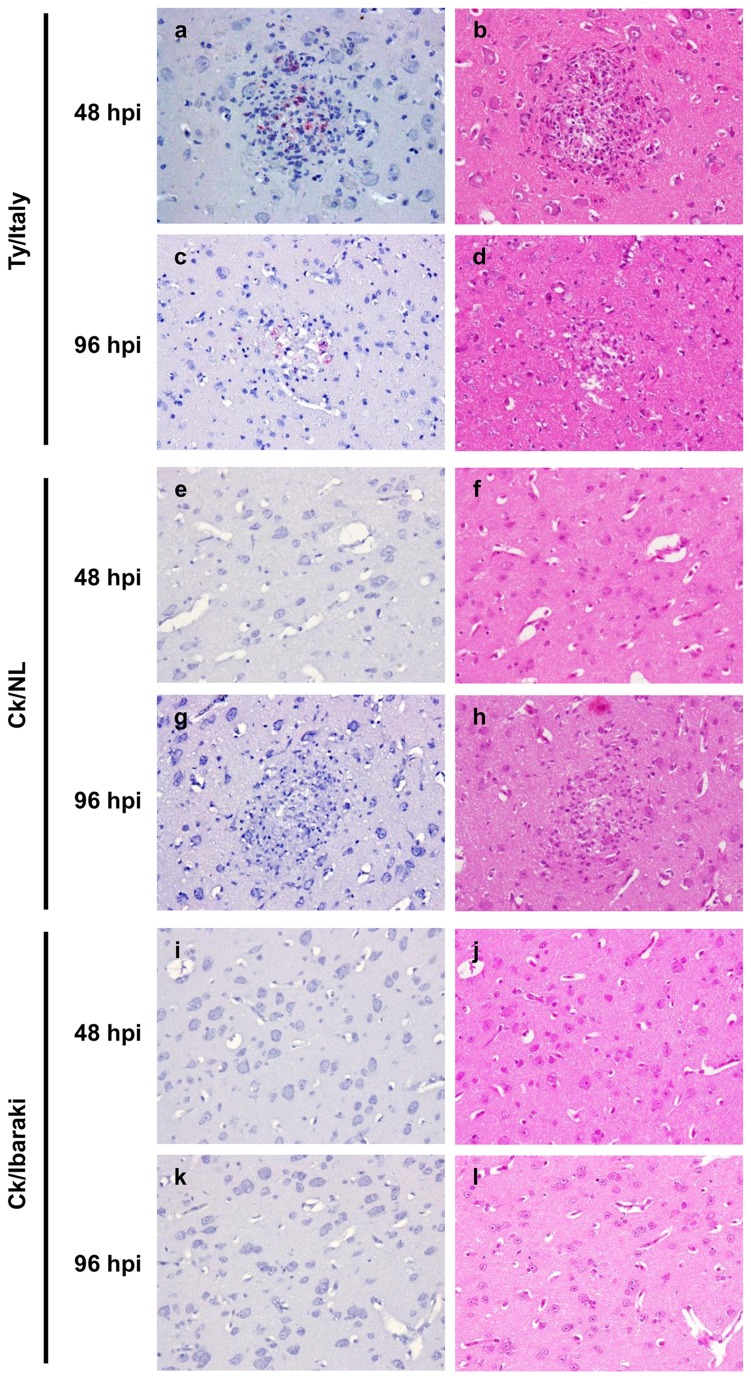
IL-6 mRNA in the brain detected using *in* *situ* hybridization. IL-6 mRNA expressing cells in the brains of the chickens infected with Ty/Italy was determined by *in situ* hybridization. IL-6 signals, represented as red color, were mainly localized to the microglial nodules on the section at 48 (**a**) and 96 hpi (**c**). The IL-6 mRNA was not detected in the brains of the Ck/NL-infected chickens (**e** and **g**), nor in those of the Ck/Ibaraki-infected chickens (**i** and **k**). The consecutive sections of HE staining (**b**, **d**, **f**, **h**, **j**, **l**) were examined by *in situ* hybridization (**a**, **c**, **e**, **g**, **i**, **k**), respectively. Original magnification, ×400.

Based on real-time PCR analyses, the IL-6 response was most significant in the brains of the Ty/Italy-infected chickens. Subsequent *in situ* hybridization identified microglial cells as the main IL-6-producing brain cells, forming nodules or scattering in the parenchyma. Consistent with PCR data, these signals with nodules were frequently found in the sections from the Ty/Italy-infected chickens obtained at 48 hpi (Figure 5a), and were rarely found in these tissues at 96 hpi (Figure 5c). On the brain section of Ck/NL-infected chickens, microglial nodules were found at 96 hpi lacking IL-6 mRNA signals (Figure 5g). No finding of microglial nodules or IL-6 mRNA positive cells was observed in the brain of the Ck/Ibaraki-infected chickens (Figure 5i and k).

### Extravasation of EB in tissues of the chickens inoculated with HPAIVs or LPAIV

**Figure 6:**
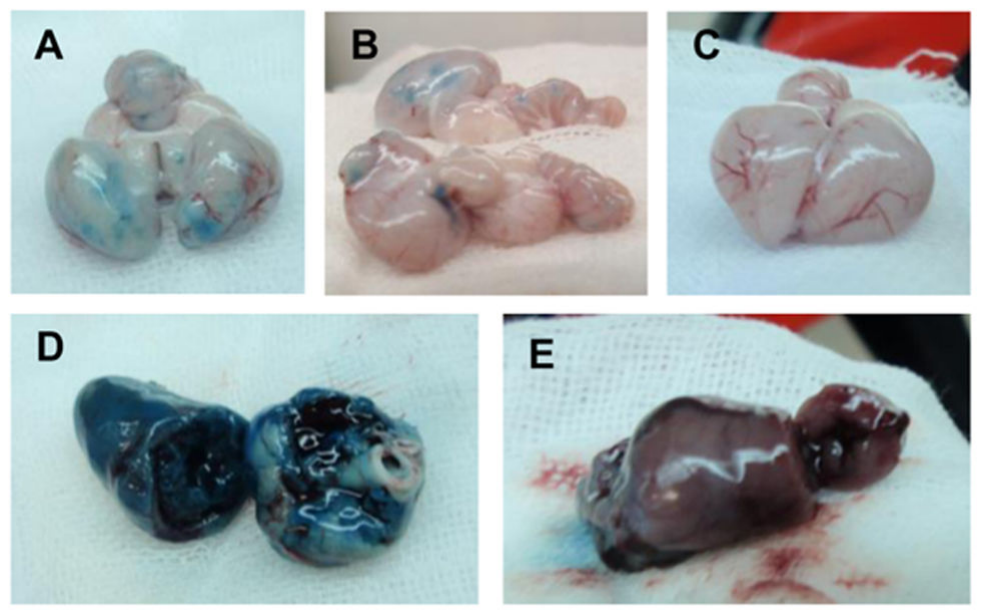
Extravasation of EB dye in the tissues of the chickens. Four days after infection, EB dye was intravenously injected into the chickens, and tissues were collected 3 h later. Photographs show brains of the chickens inoculated with Ty/Italy (**A** and **B**) or Ck/Ibaraki (**C**) and hearts of the chickens inoculated with Ty/Italy (**D**) or Ck/Ibaraki (**E**).

**Figure 7:**
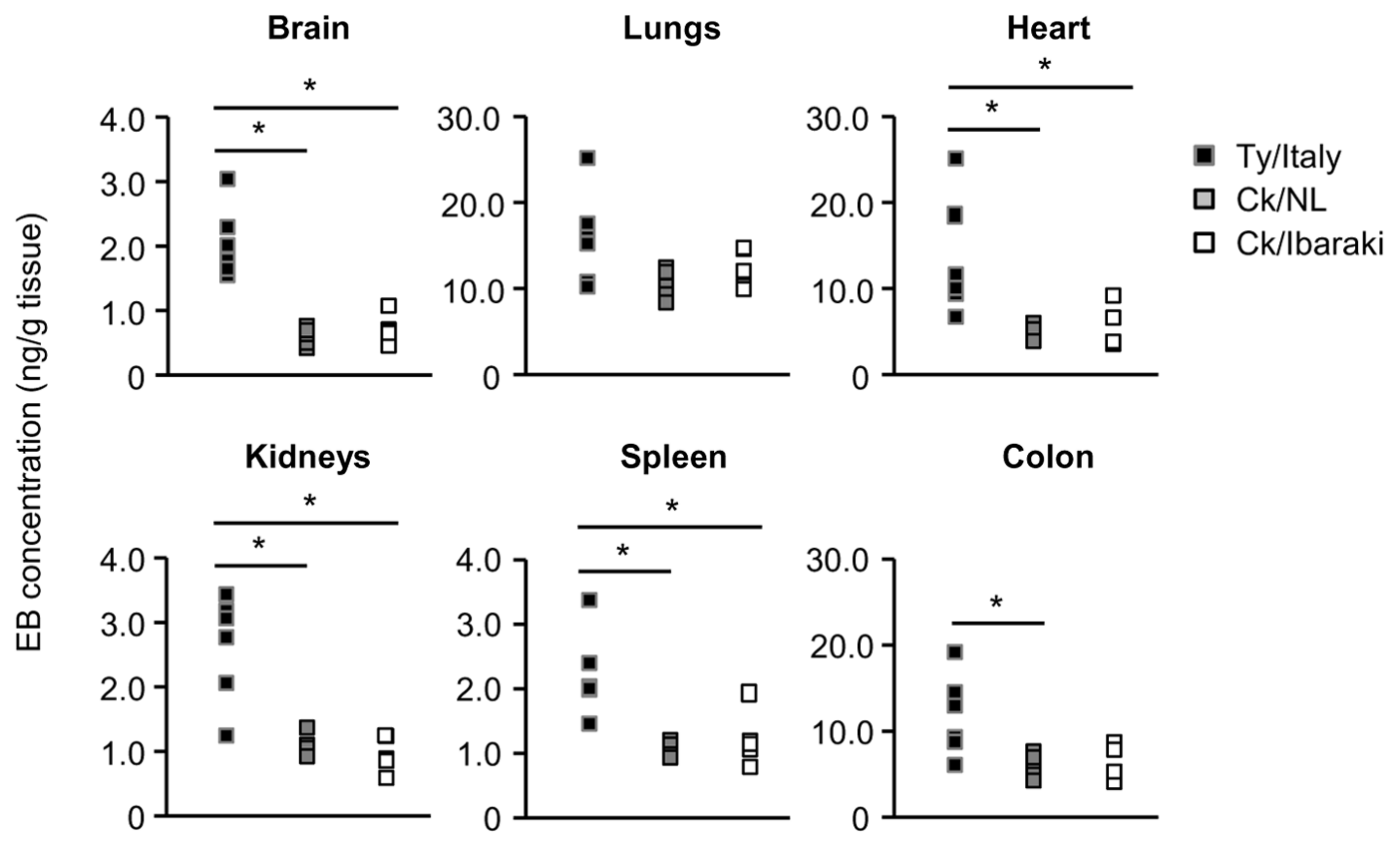
EB concentrations in the tissues of individual chickens. Values from the chickens inoculated with Ty/Italy (n = 6), Ck/NL (n = 5), and Ck/Ibaraki (n = 5) are shown with closed squares, gray squares, and open squares, respectively. The asterisk indicates a significant difference between 2 groups (P < 0.05).

To assess vascular permeability, EB dye was intravenously injected into the chickens at 4 dpi with Ty/Italy, Ck/NL, or Ck/Ibaraki. The brains (Figure 6A and B) and hearts (Figure 6D) of the chickens infected with Ty/Italy turned blue, while those of the chickens infected with Ck/NL remained normal color. Blue spotted regions were particularly prevalent in 3 of the birds infected with Ty/Italy (Figure 6A and B). The EB-stained hearts were found in 2 of the 6 chickens infected with Ty/Italy (Figure 6D), while no staining was detected in the chickens inoculated with Ck/NL. EB concentrations in the brains, hearts, spleens, kidneys, and colons of the chickens infected with Ty/Italy were significantly higher than those in the chickens infected with Ck/NL (P < 0.05; Figure 7). Based on anatomical form, the spotted region in chickens infected with Ty/Italy was identified as the choroid plexus. Of note, focal necrosis of neuronal cells, necrosis and cellular filtration into ependymal cell layers, swollen choroid plexus, thrombus formation, and hemorrhage were observed. For comparison, no extravasation of EB dye was found in the Ck/Ibaraki infected or normal chickens (Figure 6C, 6E, and Figure 7). 

## Discussion

Rapid and extensive proliferation of viruses was accompanied by rapid inflammatory and antiviral cytokine responses in tissues of the chickens inoculated with Ty/Italy, with severe capillary leakage in multiple organs during the acute phase of infection. Only moderate viral proliferation and cytokine responses were observed in the chickens inoculated with Ck/NL, and no capillary leakage was observed in any organ. Local and asymptomatic infection with the LPAIV Ck/Ibaraki did not cause significant cytokine expression or capillary leakage. These results indicate that differences in the pathogenicity of Ty/Italy, Ck/NL, and Ck/Ibaraki depend on the extent of the cytokine response, which appears proportional to the proliferation of each virus in chickens. Other reports also demonstrated the relationship between the pathogenicity of HPAIVs and cytokine responses in chickens [20–22].

Gross lesions implying vascular damage, such as hemorrhage of unfeathered skin and organ mucosa as well as edema of the face and legs, has been found in chickens infected with HPAIV [5]. In the present study, it was found that severe EB extravasation in multiple organs, thrombus formation, and hemorrhage in the brains of the chickens infected with Ty/Italy, resulting in cardiovascular abnormality and consequent multiple organ failure. Excessive cytokine responses in chickens infected with HPAIV may be a critical cause of systemic and local vascular damage. Inflammatory cytokines cause a decrease in tight junction proteins between endothelial cells, leading to hyper vascular permeability [34,35]. In addition, inflammatory cytokines such as IL-1β, IL-6, and TNF-α activate coagulation systems in infection, trauma, inflammation, and cancer [36]. Muramoto et al. [37] demonstrated that H5N1 HPAIV infection caused activation of tissue factor (TF) and coagulopathy in chickens. TF is constitutively expressed in vascular smooth muscle cells and fibroblasts, and it is produced in macrophages and endothelial cells following stimulation with inflammatory cytokines [38]. Therefore, it is strongly suggested that edema of multiple organs and coagulopathy in chickens infected with Ty/Italy should be attributed to an excessive cytokine response.

However, the association of virus proliferation and cytokine response *in vivo* has not been fully clarified. Microbial components including genomes and proteins stimulate innate immune system through pattern recognition receptors and induce various inflammatory cytokines, which affects host survival positively and negatively [39–43]. More significant cytokine response and EB extravasations were found in the brains and spleens than the lungs of the chickens infected with Ty/Italy. The difference in the cytokine mRNA expression among 3 tissues may be due to the nature of cells composing each tissue. Indeed, spleen is a secondary lymphoid organ to play a major role eliciting the immune responses, and brain cells have high capacity of producing cytokines [44,45]. The correlation of the extent of cytokine response and EB extravasation in the tissues of the chickens infected with HPAIV indicates that strong cytokine response affected host negatively rather than positively in this chicken model. It has been shown that inflammatory cytokines promote production of intracellular proteases such as matrix metalloproteinases and ectopic trypsins, which potentiate influenza virus proliferation by efficient cleavage activation of the HA [46,47].

Avian immune system has not been fully understood. Birds have a smaller repertoire of immune genes than mammals [48]. In this study, TNF-α response, which seems to be important factor for severe influenza in mammals, was less significant compared with the other cytokines in the chickens infected with Ty/Italy [14–16]. Further study will be needed to analyze the role of chicken TNF-α in the HPAIV infection since there are few papers dealing with TNF response in chickens on the infection with avian influenza virus [30].

Among the cytokines examined in this study, IL-6 showed most significant mRNA expression in the chickens infected with HPAIVs. This strong IL-6 response was also found in the livers and kidneys of the chickens infected with HPAIVs (data not shown). Marked IL-6 responses have been found in cases of severe HPAIV infection in humans and mice and in cases of influenza virus infection-associated encephalopathy [12,13,15,49,50]. IL-6 is produced by various types of cells and regulates various biological activities, including immune responses, acute and chronic inflammation, hemopoiesis, and neurotrophy [51]. An IL-6-positive feedback loop, known as the IL-6 amplifier, has been demonstrated and is characterized by the activation of NF-κB and STAT3 in type I collagen^+^ nonimmune cells. This process causes chronic inflammatory disease and transplant rejection, by leading to excessive production of IL-6 and various chemokines [52,53]. In support of these observations, IL-6 deficiency suppressed the development of autoimmune diseases in mice, and treatments with anti-IL-6 or anti-IL-6 receptor antibodies have cured these disorders in some cases [54–60]. These findings imply that IL-6 may play a pivotal role in amplifying the excessive cytokine response to influenza virus infection. Further investigations of therapeutic efficacy of IL-6 suppression during HPAIV infection in chickens are ongoing.

Although the mechanisms of influenza virus infection differ between birds and mammals, excessive cytokine responses and multiple organ failure followed by death are common to many host species [11–13,20,22]. In the present study, a strong cytokine response, especially IL-6, was accompanied by marked accumulation of antigen in the brain of the chickens infected with Ty/Italy, and microglial cells appeared to be the dominant producer of IL-6. Microglial cells are bone-marrow-derived macrophages and serve as the first defenders against infectious agents or injury-related products in the central nervous system [61]. Immunohistochemical analyses indicate that microglial cells are stimulated by virus antigen to release cytokines, including IL-6, and play a key role in driving the excessive cytokine response in chickens infected with HPAIV. Systemically, cells of the macrophage lineage are known as dominant producers of cytokines. In addition, nonimmune cells contribute to the amplification of cytokine responses in chickens infected with HPAIV, although the mechanisms of cytokine amplification have not been characterized in this model. Hence, identification of cells that trigger abnormal cytokine responses is critical to clarify the mechanisms behind excessive cytokine production and to control severe influenza. In mice, combined administration of antiviral drugs and immunomodulators has been shown to have superior efficacy than monotherapies for treatment of HPAIV infection [62]. These observations and the present findings suggest that the balance of viral proliferation and cytokine responses is important for the outcome of influenza virus infection. In this study, we have elucidated mechanisms for the severity of highly pathogenic avian influenza in chickens. These results, thus, should contribute to the development of therapeutic measures for severe cases of influenza in humans. 
